# Risks of digitalization in mental health care

**DOI:** 10.1007/s40211-025-00551-5

**Published:** 2025-10-07

**Authors:** U. Volpe, R. Ramalho, W. Gaebel

**Affiliations:** 1 Unit of Clinical Psychiatry, School of Medicine, Università Politenica delle Marche, Via Tronto 10/A, 60126 Ancona, Italy; 2 Department of Social and Community Health, School of Population Health, University of Auckland, Auckland, New Zealand; 3 WHO CC DEU-131, LVR-Klinikum Düsseldorf, Medical Faculty, Heinrich Heine University, Düsseldorf, Germany

**Keywords:** E‑mental health, Digital psychiatry, Ethical challenges, Quality assurance, Cultural safety, E‑Psychiatrie, Digitale Psychiatrie, Ethische Herausforderungen, Qualitätssicherung, Kulturelle Sicherheit

## Abstract

The digitalization of mental health care has ushered in transformative possibilities for enhancing access, diagnosis, and treatment through technologically enabled tools and platforms. However, this evolution presents substantial risks that warrant careful consideration. This paper critically examines the psychological, ethical, cultural, and clinical challenges embedded in digital mental health practices. Key concerns include diminished clinical oversight, compromised patient data security, algorithmic bias in diagnostic and therapeutic algorithms, and the erosion of traditional therapeutic relationships. Cultural disparities in digital literacy and engagement further complicate equitable care delivery. Through a multidisciplinary lens, the paper explores how these risks may impact both care outcomes and professional standards. To bridge the gap between innovation and responsible practice, a table of best practices is provided to support clinicians and developers in ethically integrating digital tools into psychiatric settings. These recommendations aim to uphold patient autonomy, strengthen clinician accountability, and preserve the humanistic foundation of mental health care. Ultimately, the paper advocates for a balanced and cautious approach to digitalization—one that embraces opportunity without compromising clinical integrity.

## Abstract (de)

Die Digitalisierung in der Psychiatrie hat transformative Möglichkeiten für die Verbesserung von Zugang, Diagnose und Therapie durch technologisch unterstützte Werkzeuge und Plattformen eröffnet. Jedoch gehen mit dieser Entwicklung substanzielle Risiken einher, die eine sorgfältige Abwägung erfordern. In der vorliegenden Arbeit werden die psychologischen, ethischen, kulturellen und klinischen Herausforderungen kritisch untersucht, die sich im Zusammenhang mit digitalen Praktiken im Bereich der psychischen Gesundheit ergeben. Zu den zentralen Punkten gehören eine verminderte klinische Übersicht, beeinträchtige Sicherheit der Patientendaten, algorithmische Verzerrung in diagnostischen und therapeutischen Algorithmen und Aushöhlung der herkömmlichen therapeutischen Beziehungen. Kulturelle Ungleichheiten hinsichtlich digitaler Kompetenz und Engagement verkomplizieren gerechte Gesundheitsversorgung noch mehr. Aus multidisziplinärer Sicht wird in der vorliegenden Arbeit untersucht, wie diese Risiken sowohl Therapieergebnisse als auch Berufsstandards beeinflussen können. Zur Überbrückung der Kluft zwischen Innovation und verantwortungsvoller Praxis ist eine Tabelle der besten Vorgehensweisen zur Unterstützung von klinisch Tätigen und Entwicklern bei der ethisch vertretbaren Integration von digitalen Werkzeugen in psychiatrische Einrichtungen enthalten. Ziel der darin enthaltenen Empfehlungen ist die Aufrechterhaltung der Patientenautonomie, Stärkung der Verantwortung der klinisch Tätigen und Erhaltung der humanistischen Grundlagen der Psychiatrie. Schließlich wird in der vorliegenden Arbeit eine ausgewogener und vorsichtiger Ansatz bei der Digitalisierung befürwortet – einer, der die Gelegenheit ergreift, ohne die klinische Integrität zu beeinträchtigen.

## Introduction

Digitalization has significantly altered the landscape of health care organization and delivery, particularly in psychiatry. While mental illnesses are often underdiagnosed and undertreated, several obstacles help explain this public health problem, including provider/psychiatric workforce shortage, difficulty accessing care, cost, stigma, and a variety of diagnosis-specific issues. By promising to broaden access, increase efficiency, decrease costs, and remove stigma, digital tools for mental health have been touted as a viable solution. Digital psychiatry indeed offers significant advantages in clinical care by addressing current barriers such as limited access to mental health services, long waiting times, stigma and geographic disparities [1]. Through telepsychiatry, mobile apps, and digital monitoring tools, clinicians can extend care to currently underserved populations, improve continuity of treatment, and facilitate real-time symptom tracking [2]. This may not only enhance patient engagement and early intervention but also allow for more data-informed and hence personalized care. As the demand for mental health services continues to outpace supply, digital solutions may represent a scalable and efficient complement to traditional care [3]. Although recent research has shown that digital tools contribute to making mental health services more accessible and scalable, these innovations do not come without substantial risks [4]. For example, unlike somatic medicine, psychiatry heavily depends on nuanced interpersonal dynamics and subjective experiences that may be difficult to digitalize. Presented here are the multifaceted ‘dangers’ of digitalization in psychiatric care, drawing on clinical, social, ethical, and technical perspectives. To help move this conversation forward, this paper critically examines the risks of digital psychiatry across five domains: clinical, ethical, sociocultural, technological, and regulatory.

## Clinical risks

Digital platforms often rely on symptom checklists and structured inputs, potentially missing the nuanced assessments a clinician makes in person. As a result, diagnostic tools embedded in mental health apps or online platforms may oversimplify complex psychiatric conditions [5]. Furthermore, algorithmic models trained on limited datasets can misdiagnose or overlook comorbidities. Also, nonverbal cues (e.g., body language, affect expressivity) may be poorly conveyed over video, and some patients may feel emotionally distant from clinicians in virtual settings. Thus, while telepsychiatry has increased access to care, especially during the COVID-19 pandemic, it may undermine the quality of care for certain populations, and the risk of an overreliance on telepsychiatry cannot be ruled out at the present moment [6].

The therapeutic alliance is foundational to psychiatric care and some concerns about its deterioration in a digital setting have been recently put forward. Especially asynchronous digital mediums (e.g., chatbots or self-help apps) may weaken this bond, leading patients to feel less understood or supported, potentially reducing adherence to treatment [7].

## Ethical risks

One classical concern leading to the “digital divide” in the health care domain is that of handling sensitive data in terms of individual privacy and, more generally, of data surveillance. Mental health apps and algorithms frequently collect sensitive data such as mood logs, sleep patterns, geolocation and social interactions. Studies have shown that many apps do not comply with standard privacy practices or inform users about data sharing with third parties [8]. Of course, data breaches can have severe consequences in general, but given the stigma surrounding mental illness, consequences in this field may be even worse. This risk is made particularly high, given that digital mental health tools often present lengthy, opaque terms of service that patients may not fully understand. This may significantly challenge the principle of informed consent [9], especially in vulnerable populations, such as adolescents or those with psychotic disorders, whose ability to make autonomous decisions regarding digital engagement is further diminished. Trachsel and Sedlakova [10] recently argued that these ethical challenges highlight the importance of respecting patient autonomy, not only in terms of data consent but also in allowing patients to actively choose (if available) between digital and face-to-face psychiatric care. Failure to provide patients with this choice, especially when digital alternatives are less transparent or secure, risks compromising their autonomy and eroding trust in mental health services. Finally, the rapid development of digital mental health technologies often outpaces existing regulatory frameworks: most apps are not yet classified as medical devices and therefore bypass rigorous validation processes. While some regulatory bodies in the USA, in the EU or in single countries (e.g., Germany, UK) have proposed recommendations or implemented specific regulations for medical apps, the global landscape remains largely unregulated. As a result, clinicians and patients must navigate a marketplace where efficacy and safety are not guaranteed, posing a significant risk due to insufficient regulatory oversight [11].

## Sociocultural risks

Not all patients may have equal access to digital tools. Socioeconomic disparities, digital literacy gaps, and regional infrastructural deficiencies can exacerbate existing health inequities [12]. For example, rural populations and the elderly—although having equal access potential—may lack the technological fluency to engage with telepsychiatry. This classical example of equality (i.e., giving everyone the same resources or opportunities) vs. equity (i.e., individuals have different needs and circumstances, and therefore may require different resources or support to achieve the same outcome) applies relevantly to the field of digital psychiatry and mitigating solutions to ensure equity in digital mental health care are still needed.

Moreover, as most digital mental health platforms and software are developed in Western contexts, the risk of making them not culturally responsive or ineffective for non-Western users is highly relevant. Standardized assessments may not capture culturally specific expressions of distress or coping mechanisms of specific contexts, leading to a substantial cultural incongruity of digital tools for mental health care [13]. In psychiatry, it is essential to create therapeutic environments that respect and acknowledge patients’ cultural identities, values, and lived experiences. In general, doctors’ cultural competence is essential to ensure the patient feels emotionally and culturally safe during treatment. However, in psychiatric care, misdiagnosis or misunderstanding can more easily arise from cultural disconnects. Thus, ensuring cultural safety is essential to ensure both ethical and effective standards of practice. Clinicians must engage in continuous self-reflection to recognize one’s limits and adapt interventions to meet the cultural perspective of diverse populations [14].

## Technology-related risks

Ironically, the very tools meant to alleviate mental distress may induce or exacerbate it. Continuous self-monitoring through mental health apps can heighten anxiety, leading to a preoccupation with symptoms [15]. Additionally, interaction with artificial intelligence (AI)-driven platforms may foster feelings of depersonalization and isolation, with possible mediation effect of the sense of self-efficacy [16]. Also, digital diagnostic tools and AI models can perpetuate biases present in training data. If algorithms are trained on datasets that underrepresent minority populations, the tools may yield less accurate results for these groups, leading to “diagnostic injustice” [17]. This can erode trust in mental health systems and worsen disparities. Finally, a recent metanalysis [18] also pointed out that—although such phenomenological entities have not been officially included in current diagnostic systems—“digital addictions” represent a growing phenomenon. Its global pooled prevalence of more than 25% of the general population and the significant variations in terms of geographic distribution and type of digital tool highlight that these phenomena deserve greater attention in mental health care settings.

## Conclusion

The digital transformation of psychiatric and mental health care offers unprecedented opportunities to improve access, personalization, and efficiency of treatment. However, its rapid and often uncritical adoption brings with it a host of complex risks. While not an exhaustive discussion, this paper presented a necessary overview of these clinical, ethical, sociocultural, and technology-related risks, These include clinical misapplications, ethical oversights, algorithmic biases, and the potential weakening of the therapeutic alliance—an essential cornerstone of psychiatric care. Such risks are not merely theoretical; they have practical implications that can compromise patient outcomes, particularly among vulnerable populations. Therefore, the integration of digital tools into mental health practice must be approached with thoughtful regulation, ongoing clinical evaluation, and sensitivity to cultural and contextual differences. Table 1 offers a synthesized overview of the most frequent pitfalls encountered in the clinical use of digital mental health tools, along with a set of preliminary best practice guidelines grounded in current empirical evidence. These are intended as a starting point for clinicians and stakeholders seeking to adopt digital innovations responsibly and effectively.

**Table 1:** Best practice examples to overcome risks and potential ‘risks’ in digital psychiatry

Do’s	Don’ts
Vet digital tools for evidence-based validity	Rely solely on artificial intelligence (AI)- or app-based diagnoses
Ensure transparent, readable privacy policies	Use platforms that collect data without consent
Maintain blended care (digital + human)	Replace therapeutic relationships with chatbots
Incorporate cultural safety in tools	Assume one-size-fits-all models work universally
Provide training for clinicians and patients	Ignore digital literacy and accessibility barriers
Regularly evaluate outcomes and update tools	Use outdated or unverified applications
Protect data with robust cybersecurity	Store sensitive mental health data insecurely

Looking ahead, meaningful progress will depend on sustained collaboration between mental health professionals, individuals with lived experience, digital health developers, ethicists, and policymakers. Only through interdisciplinary dialogue and cocreation can we ensure that digital psychiatry develops not as a substitute for human care, but as a tool that reinforces and enriches it—preserving core clinical values while embracing technological innovation.
